# Inhibition of αvβ3 integrin impairs adhesion and uptake of tumor-derived small extracellular vesicles

**DOI:** 10.1186/s12964-020-00630-w

**Published:** 2020-09-25

**Authors:** Wanessa F. Altei, Bianca C. Pachane, Patty K. dos Santos, Lígia N. M. Ribeiro, Bong Hwan Sung, Alissa M. Weaver, Heloisa S. Selistre-de-Araújo

**Affiliations:** 1 Biochemistry and Molecular Biology Laboratory, Department of Physiological Sciences, Federal University of São Carlos, São Carlos, Brazil; 2 Department of Biochemistry and Tissue Biology, Institute of Biology, State University of Campinas-UNICAMP, Campinas, São Paulo Brazil; 3 Department of Cell and Developmental Biology, Vanderbilt University School of Medicine, Nashville, USA; 4 Department of Pathology, Microbiology, and Immunology, Vanderbilt University Medical Center, Nashville, USA

**Keywords:** Small extracellular vesicles, αvβ3 integrin, Adhesion, Uptake, Breast cancer

## Abstract

**Background:**

Extracellular vesicles (EVs) are lipid-bound particles that are naturally released from cells and mediate cell-cell communication. Integrin adhesion receptors are enriched in small EVs (SEVs) and SEV-carried integrins have been shown to promote cancer cell migration and to mediate organ-specific metastasis; however, how integrins mediate these effects is not entirely clear and could represent a combination of EV binding to extracellular matrix and cells.

**Methods:**

To probe integrin role in EVs binding and uptake, we employed a disintegrin inhibitor (Dis*Ba*-01) of integrin binding with specificity for αvβ3 integrin. EVs were purified from MDA-MB-231 cells conditioned media by serial centrifugation method. Isolated EVs were characterized by different techniques and further employed in adhesion, uptake and co-culture experiments.

**Results:**

We find that SEVs secreted from MDA-MB-231 breast cancer cells carry αvβ3 integrin and bind directly to fibronectin-coated plates, which is inhibited by Dis*Ba*-01. SEV coating on tissue culture plates also induces adhesion of MDA-MB-231 cells, which is inhibited by Dis*Ba*-01 treatment. Analysis of EV uptake and interchange between cells reveals that the amount of CD63-positive EVs delivered from malignant MDA-MB-231 breast cells to non-malignant MCF10A breast epithelial cells is reduced by Dis*Ba*-01 treatment. Inhibition of αvβ3 integrin decreases CD63 expression in cancer cells suggesting an effect on SEV content.

**Conclusion:**

In summary, our findings demonstrate for the first time a key role of αvβ3 integrin in cell-cell communication through SEVs.

Video Abstract

**Graphical abstract:**

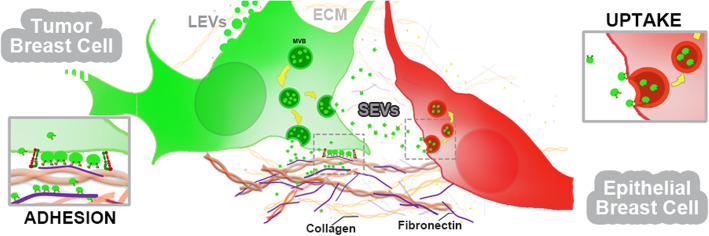

## Background

During tumor progression, cancer cells and neighboring host cells interact with each other and with extracellular matrix (ECM) proteins including collagen, laminin, vitronectin, and fibronectin [1]. Because ECM influences cell polarity, migration, proliferation, differentiation and survival, its disorganization during cancer progression facilitates cellular transformation, metastasis and may contribute to drug resistance [2, 3].

Cells are capable of transferring information to other cells and to the microenvironment via EVs [4, 5]. EVs are membranous nanoparticles secreted from cells that carry a variety of bioactive molecular cargoes such as nucleic acids, proteins, and lipids [6–8]. EVs are currently classified into different subtypes according to their size, biogenesis mechanisms, cargoes, and so on [9]. They include larger EVs (LEVs) such as shed microvesicles (100–1000 nm) from the cell membrane, [7] and SEVs, which include exosomes (50–150 nm) secreted from endocytic pathways [10, 11].

Integrins are critical adhesion receptors for ECM proteins that support cell adhesion and drive cell migration. For example, α1β1 and α2β1 integrins are major collagen receptors, whereas fibronectin binds preferentially to α5β1 and αvβ3 integrins. Amongst the particular sequences recognized by integrins, the RGD (Arg-Gly-Asp) motif is found in many ECM proteins including vitronectin, fibronectin and laminin [12]. Although RGD is usually recognized by both α5β1 and αvβ3 integrins, these two integrins play divergent roles in cell adhesion and migration. Fibronectin adhesion by α5β1 integrin results in highly dynamic thin cell protrusions in multiple directions while adhesion to αvβ3 integrin results in a single large lamellipodium with more static adhesions at the leading edge [13–15]. In addition to the RGD motif, integrin and ECM conformations are crucial to their interaction, indicating a complex mechanism [16, 17].

During tumor development, expression levels of integrins change in response to alterations on ECM [18]. αvβ3 integrin is highly expressed in aggressive cancers, which is related to increase of tumor cell migration, adhesion and invasion during tumor progression [13, 19–23]. Since integrin inhibition blocks cell migration, these receptors were considered a valuable target on cancer research [15, 16, 24, 25]. Cilengitide, a cyclic RGD-containing peptide that antagonizes αvβ3 and α5β1 integrins at nanomolar ranges had promising preclinical results but it was ineffective in phase I clinical outcomes [16].

As an alternative to RGD peptides, disintegrins, a family of cysteine-rich peptides, present anti-migratory and anti-angiogenic effects in tumors with non-toxic properties [26, 27]. Originally found in snake venom, most of the disintegrins contain the adhesive RGD motif and are potent antagonists of αvβ3 and α5β1 integrins. Dis*Ba*-01 is a recombinant His-tag fusion RGD-disintegrin from *Bothrops alternatus* snake venom and a selective nanomolar αvβ3 integrin inhibitor. Firstly recognized by its anti-platelet and anti-thrombotic effects [27, 28], this protein also decreased migration speed and directionality of oral carcinoma cells [15]. We have demonstrated that Dis*Ba*-01 affinity for αvβ3 integrin (K_d_ 4.63 × 10^− 7^ M) is one hundred times higher than its affinity for α5β1 integrin (K_d_ 7.62 × 10^− 5^ M), which makes this protein an excellent tool to study the roles of αvβ3 integrin in the adhesion and migration processes [15, 16, 27].

Integrins carried on SEVs have been shown to support tumor spread and metastasis development [18, 29–33] while exosomes mediate cell adhesion to matrix components [34, 35]. In addition, α5β1 integrin found on exosomes was shown to bind fibronectin and promote cancer cell adhesion and motility [36]. αvβ3 integrin present on SEVs from PC3 and CWR22Pc prostate cancer cells induced migration of non-tumorigenic BHP-1 cells [30]. Moreover, in vivo studies showed the transfer of αvβ3 integrin from SEVs to β3-negative recipient cells resulting in acquired ligand binding activity of the recipient cells [37]. In metastasis, integrins carried by exosomes from lung tropic models have been associated with organ site-specific metastasis, including α6β4 and αvβ5, which are associated with metastasis in lung and liver tissues, respectively [29, 38].

Despite the aforementioned data, the real contribution of EV-carried integrins to cellular communication in tumor development is still unclear. To better understand how αvβ3 integrin receptors work in the context of EVs, we investigated the effect of integrin blocking on SEV adhesion and uptake by using the recombinant disintegrin Dis*Ba*-01. We show that Dis*Ba*-01 inhibits αvβ3 integrin on isolated SEVs, affecting their adhesion to purified ECM proteins and their uptake in recipient cells. Moreover, the treatment of Dis*Ba*-01 to MDA-MB-231 cells expressing GFP-CD63 affects intracellular GFP-CD63 expression suggesting an effect on SEV cargo sorting. As far as we know, this is the first report of such roles for EV-carried αvβ3 integrin and it further supports a key role for integrins in SEV recognition and uptake by recipient cells.

## Methods

### Cell lines and culturing

MDA-MB-231 (malignant) and MCF 10A (non-malignant) breast cell lines were purchased from ATCC and maintained in DMEM (Dulbecco’s Modified Eagle Medium) and DMEM-F12, respectively. DMEM was supplemented with 10% fetal bovine serum (FBS) and DMEM-F12 was supplemented with 5% horse serum (HS). In experiments using SEVs, culture media were supplemented with SEV depleted serum (SEV^−^). To prepare FBS SEV^−^ and HS EV^−^, the sera were ultracentrifuged at 100,000 x g overnight and the supernatant was collected. Cells were cultured at 37 °C in 5% of CO_2_ atmosphere. Cell number was counted and cell viability was verified in a TC20 automated cell counter (Bio-Rad, Hercules, CA, USA) with 0.4% trypan blue (Thermo Scientific, Waltham, MA, USA) prior to experiments.

pLenti-GFP-CD63 plasmid, previously described by Hoshino et al. [39], was used to make MDA-MB-231 cells stably expressing GFP-CD63. Human MDA-MB-231 cells (ATCC) and 293 FT packaging cells were grown in DMEM + 10% FBS. 293 FT cells transfection, viral harvest, and transduction of MDA-MB-231 cells were performed as previously described [40]. Transduced cells were selected with 4 μg/ml of puromycin for 7 days.

### Integrin inhibitor

Recombinant Dis*Ba*-01 was isolated from inclusion bodies of *E. coli* BL21(DE3)-pET28a-Dis*Ba*-01 culture and purified to homogeneity as previously described [27]. Purified disintegrin was labeled with Alexa Fluor 546 (Invitrogen) according to the manufacturer’s instructions.

### Isolation and purification of EVs from conditioned media

For EV isolation, 2.0 × 10^6^ MDA-MB-231 cells were plated in T-150 flasks containing 15 ml of culture media (total = 10 flasks) and cultured for 48 h until 80% of confluence. The culture media was replaced with 15 ml of Opti-MEM and cells were further cultured for 48 h to obtain conditioned media. Conditioned media was submitted to serial centrifugation to sediment live cells (300 x g for 10 min), dead cells (2000 x g for 25 min), and large EVs (10,000 x g, Ti40 rotor for 30 min), respectively. The supernatant was concentrated to 30 ml volume in a concentrator (Sartorius, VS6041Cat# and 100 k MWCO), layered over 2 ml of 60% iodixanol in an ultracentrifuge tube (25 × 89 mm for SW 32 Ti rotor), and further centrifuged at 100,000 x g for 4 h. We collected 3 ml from the bottom of the tube and layered it in a new centrifuge tube (40% iodixanol). Iodixanol solutions (20% wt/vol, 10% wt/vol, and 5% wt/vol) were layered over from the bottom to the top. Iodixanol solutions were prepared by diluting OptiPrep (60% wt/vol aqueous iodixanol; Axis-Shield PoC) with 0.25 M sucrose / 10 mMTris, pH 7.5. The gradient was centrifuged at 100,000 x g for 18 h using a SW45 Ti rotor and 12 fractions of 1 ml each were collected. Two ml of PBS was added to 1 ml fractions and ultracentrifuged in a tabletop centrifuge at 100,000 x g for 3 h using a TLA 100.3 rotor. Vesicles were resuspended in 50 μl of PBS for subsequent studies. Purified LEVs and SEVs were quantitated by Particle Metrix ZetaView PMX 110 and the protein amount was measured using microBCA Protein Assay Kit (Thermo Fisher 23,235).

We also uploaded all relevant data of our experiments to the EV-TRACK knowledgebase (EV-TRACK ID: EV190006) [41].

### Characterization of purified EVs

#### Transmission electron microscopy

For negative staining of purified SEVs*,* 5 μl of EV samples was added to Formvar carbon film-coated grids (FCF-200-Cu; Electron Microscopy Sciences; Hatfield, PA) for 60 s. Grids were immediately fixed with 4% paraformaldehyde in water for 20 min, stained with 2% uranyl acetate for 2 min, and allowed to air-dry. For each step, the excess of solution was removed by wicking with a filter paper. The grids were imaged using a TEM Tecnai F20 G2, 200Kv in 40,000 x magnification.

#### Western blotting

Purified EVs were lysed with 1% SDS 50 mM Tris pH 7.6-lysis solution, mixed with SDS sample buffer, and loaded onto 8% acrylamide gels (10 μl). Gels were transferred to nitrocellulose membranes (0.45 μm, Biorad) and blocked with 5% bovine serum albumin (BSA) in Tris-buffered saline with 0.05% Tween 20 (TBS-T) for 1–2 h. Membranes were probed with antibodies for EV markers, anti-CD63 (1:1000, Abcam, ab59479), anti-Flotillin (1:1000, BD, 610821), and anti-Alix (1:1000 Sigma, SAB 4200476). As a negative control, anti-Calnexin (1:1000, Cell Signaling, mAb 2679) was used. Appropriate secondary antibodies were added and detected by ECL (Thermo Scientific, 32,106 and 34,095). The same procedure was applied to detect integrins and ECM proteins such as fibronectin (Abcam, ab2413) and collagen (Abcam, ab34710).

### Adhesion of isolated SEVs to different ECM proteins

Ninety-six well plates were coated with collagen (10 μg /ml) or fibronectin (2 μg /ml) overnight at 4 °C. For the experiment, isolated SEVs were labeled with ExoGlow (System Bioscience Uniscience) according to the manufacturer’s instructions. Prior to incubation, vesicles were incubated with Dis*Ba*-01 in different concentrations (250, 500 and 1000 nM) in ice for 30 min and then plated (1.0 × 10^8^ vesicles/well) over the coating for 4 h. After incubation, non-adherent SEVs were washed out and photomicrographs were acquired using a Nikon Plan Apo 60x/1.40 NA oil immersion lens in a Nikon Eclipse TE2000E microscope. For fluorescence intensity analysis, adhered green fluorescent vesicles were segmented from the background by thresholding and measured for integrated intensity using ImageJ Fiji (Analyze tab/Measure).

### Cell adhesion on EV coating

Isolated small (EVs obtained from 100,000 x g ultracentrifugation for 18 h, Suppl. Figure 1) or large EVs were resuspended in PBS and added to a 96 well plate overnight (50 μg /ml). Prior to adhesion experiments, the wells were blocked with 1% BSA for 1 h. Fibronectin-coated wells (10 μg/ml) were used as a positive control of cell adhesion. Dis*Ba*-01 (100 nM) was incubated on EV coating for 30 min. After washing unbound Dis*Ba*-01 using PBS, calcein-labeled MDA-MB-231 cells (5 × 10^6^) were added and allowed to attach for 1 h. To measure adhesion, green fluorescence intensity was read in a SpectraMax I3 (Molecular Devices) plate reader.

### SEV uptake by healthy breast cells

#### Uptake of purified SEVs

One day before the experiment, MCF 10A cells were plated in a 96 well plate in a density of 2.5 × 10^3^ cells/well. Small EVs were labeled with ExoGlow kit (System Biosciences) and subsequently treated with Dis*Ba*-01 (100 or 1000 nM). After treatment with the integrin inhibitor, 1.4 × 10^7^ vesicles/well were added over the MCF 10A cells and allowed to internalize for 4 h. After incubation, the supernatant was removed and cells were washed. The uptake of ExoGlow-labeled SEVs was analyzed by epifluorescence microscopy in the automated system ImageXpress Micro XLS (Molecular Devices) using a Nikon S Plan Fluor ELWD 40X /0.60 NA magnification lens.

#### Co-culture in a transwell system

MCF 10A cells (1 × 10^3^) were plated on glass coverslips inside a 24-well plate. After MCF 10A cell adhesion, transwell inserts with pore size of 0.4 μm were added to the wells. MDA-MB-231 cells expressing GFP-CD63 (1 × 10^4^, treated and non- treated with Dis*Ba*-01 at 1000 nM for 30 min prior incubation) were added to the transwell inserts and incubated for 6 h. After incubation, MCF 10A cells were stained with DAPI and the cytoplasm marker Cell Tracker Red CMTPX according to the manufacturer instructions (Invitrogen, C34552). MCF10A cells were imaged in a Zeiss LSM 780 confocal microscope using a 63 X/1.3 NA objective lens.

To measure the vesicles in MCF 10A cells, the green fluorescence was quantitated by integrated intensity analysis. Percentage of inhibition was calculated by comparing the integrated intensity of green fluorescence in MCF 10A cells incubated with Dis*Ba*-01-treated MDA-MB-231 cells expressing GFP-CD63 to that incubated with Dis*Ba*-01-non-treated MDA-MB-231 cells expressing GFP-CD63.

### Imaging for co-culture

#### Fluorescence microscopy

MCF 10A cells (1.5 × 10^5^) were cultured on glass coverslips for 48 h. After adhesion, cells were labeled with Cell Tracker Red CMTPX according to the manufacturer instructions (Invitrogen, C34552). MDA-MB-231 cells (1.0 × 10^6^) labeled with Cell Trace CFSE (Life Technologies, C34554) according to the manufacturer instructions or stably expressing GFP-CD63 were treated with 100 nM or 1000 nM of Dis*Ba*-01 for 30 min (untreated cells were used as a control), plated over an MCF 10A monolayer and incubated for 24 h. Cells were fixed with 4% paraformaldehyde and stained with DAPI. Z-stack images were acquired in a Zeiss LSM 880 with Airyscan confocal microscope, using a 63x/1.40 Plan-APOCHROMAT oil lens and quantitated by integrated intensity using Fiji (Analyze tab/Measure). For extracellular SEV quantification, cell bodies were carefully selected and deleted from each image. GFP-CD63 deposits surrounding cells were segmented from the background by thresholding and measured for area and integrated intensity using Fiji (Analyze tab/Measure). For cell morphology measurements, each cell (ten cells per experiment) was manually selected and segmented from the background by thresholding and measured for area using Fiji (Analyze tab/Measure).

#### Scanning electron microscopy (SEM)

MCF 10A cells (2.0 × 10^4^) were plated in a Lab-TeK® chamber slide™ (LOBOV, Catalog Number: 177402) and incubated at 37 °C overnight. On the next day, MDA-MB-231 cells (Dis*Ba-*01-treated or non-treated) were plated at the same density over the MCF 10A cells and allowed to adhere for additional 24 h. Cells were then washed with PBS, fixed with 4% glutaraldehyde (Sigma-Aldrich®) for 1 h at 37 °C, and dehydrated by increasing ethanol concentration (50, 60, 70, 80, 90 and 100%, 10 min for each steps) before drying with hexamethyldisilazane (Sigma-Aldrich®). Cell morphology was characterized by scanning electron microscopy (Inspect F50 - FEI®). Cell morphology quantitation was performed by measuring the area of cells, using Fiji (Analyze tab/Measure).

### Dis*Ba*-01 uptake assay

Dis*Ba*-01 was labeled using Alexa Fluor® 546 dye (Invitrogen, Thermo Scientific) according to the manufacturer’s instructions. MDA-MB-231 expressing GFP-CD63 cells (1 × 10^4^) were plated in 8-well Nunc™ Lab-Tek™ Chamber Slide (Thermo Scientific) and incubated overnight. On the next day, cells were incubated with Dis*Ba*-01 (1000 nM) for 1 h and 4 h, fixed with 4% paraformaldehyde, and stained with DAPI. Slides were mounted with mounting media Prolong™ (Thermo Fisher Scientific). Confocal images were acquired in a Zeiss LSM 780 confocal microscope using a 63 X/1.3 NA objective lens. Zen software was used to acquire images and laser power was the same for all conditions in order to compare the fluorescence intensity between different conditions.

### Data analysis and statistics

At least three independent experiments were performed to acquire data for quantitation. All data sets were tested for normality using Shapiro-Wilk normality test in GraphPad Prism software. Mean ± standard error of the mean (SEM) were calculated and intergroup comparisons were made using One-way ANOVA or *t* Test (two-tailed paired or unpaired with Welch’s correction) analysis. Values of *p* < 0.05 were considered statistically significant.

## Results

### Isolation, purification and characterization of SEVs from MDA-MB-231 cells

**Fig. 1:**
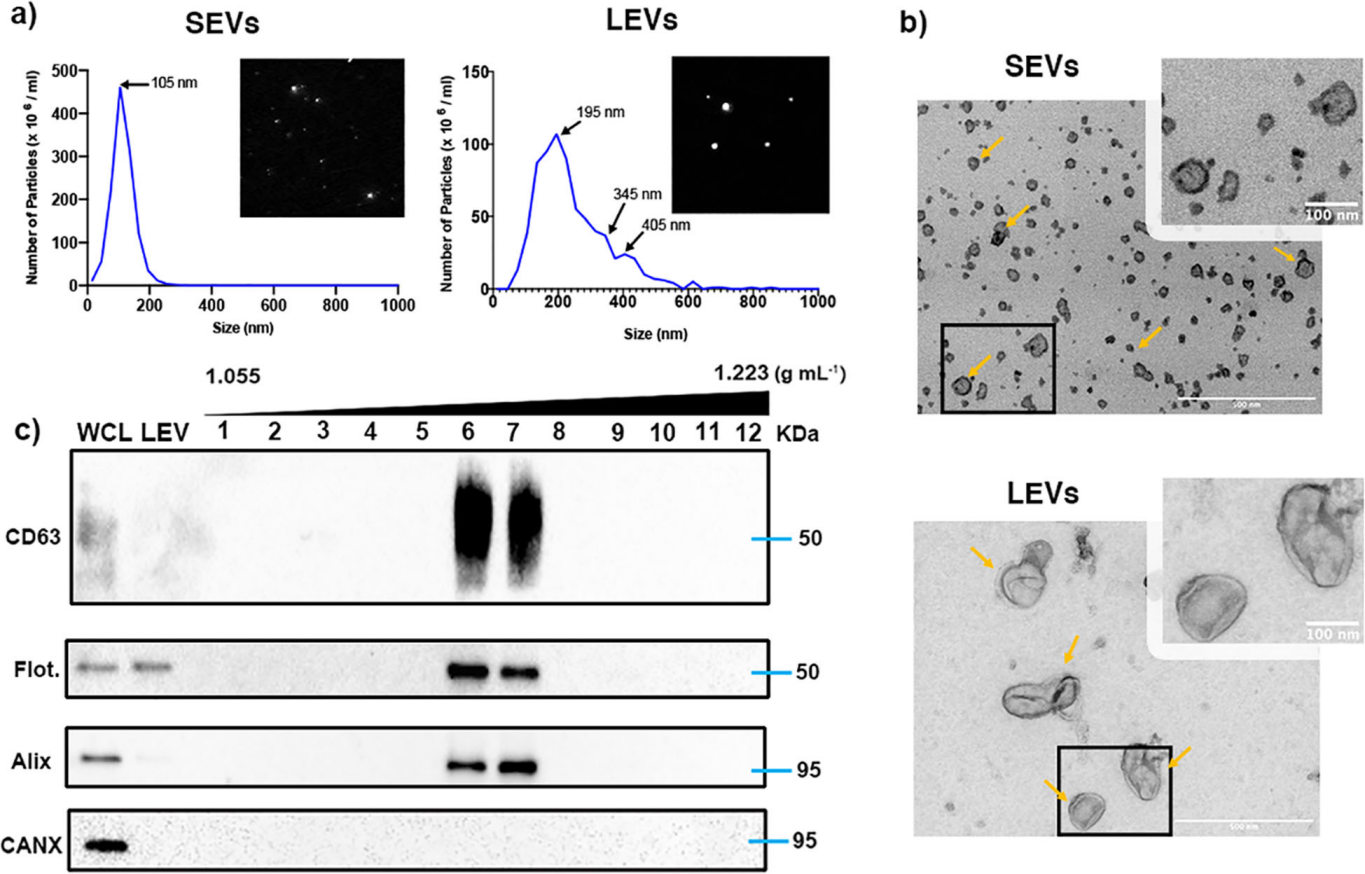
Validation of cancer cell-derived EV isolation and characterization. **a** Representative trace and video acquisition snapshot from nanoparticle tracking analysis of small and large EVs. Both traces show vesicles within a typical size profile. **b** Transmission electron microscopy of density gradient-purified SEVs and LEVs. Yellow arrows point to representative EVs. Scale bar: 500 nm (large image) and 100 nm (zoomed images). **c** Western blotting for the EV markers CD63, Flotillin and Alix across 12 iodixanol density gradient fractions, large vesicles (LEV) and whole cell lysate (WCL) of the EV donor cells. Calnexin (CANX) antibody was used as a negative control

EVs were obtained from high-speed differential centrifugation of MDA-MB-231 cells conditioned medium. Considering the effect of serum-free conditions on cell health, the viability of EV-producing MDA-MB-231 cells was tested prior SEVs isolation to assure good quality of cells at the moment of medium collect. LEVs were collected by centrifugation at 10,000 x g for 30 min. SEVs present on supernatant were concentrated onto a cushion of iodixanol and further purified by iodixanol density gradient ultracentrifugation [42]. Purified EVs were characterized by Western blotting, particle size analysis and transmission electron microscopy (TEM) (Fig. 1). Nanoparticle tracking analysis (NTA) showed vesicles in the size range for typical SEVs with a peak at 110 nm and LEVs with peaks at 195, 345, and 405 nm (Fig. 1a), consistent with previous descriptions [11]. SEV size and morphology were confirmed by TEM (Fig. 1b). Western blot analysis identified SEVs in fractions 6 and 7 of the gradient enriched with the SEV markers CD63, flotillin, and Alix, and LEVs positive for flotillin, while the negative control calnexin was detected only in the cell lysate (Fig. 1c). These data, together with NTA and TEM analysis, indicate that the purified SEVs preparation has typical characteristics of SEVs [7, 43–45].

### Purified SEVs bind to extracellular matrix proteins and support cell adhesion through αvβ3 integrin binding

Integrins carried by EVs should be displayed on the outside of the vesicles, competent to interact with ECM. To identify the ability of SEVs to adhere to ECM, purified SEVs were labeled with ExoGlow kit (System Biosciences) and added to tissue culture dishes coated with purified ECM components. As the ExoGlow dye is based on the carboxyfluorescein succinimidyl diacetate ester (CFSE) chemistry, upon internalization into intact EVs, it is hydrolyzed to a fluorescent green structure, allowing the employment of EVs on in vitro assay.

**Fig. 2:**
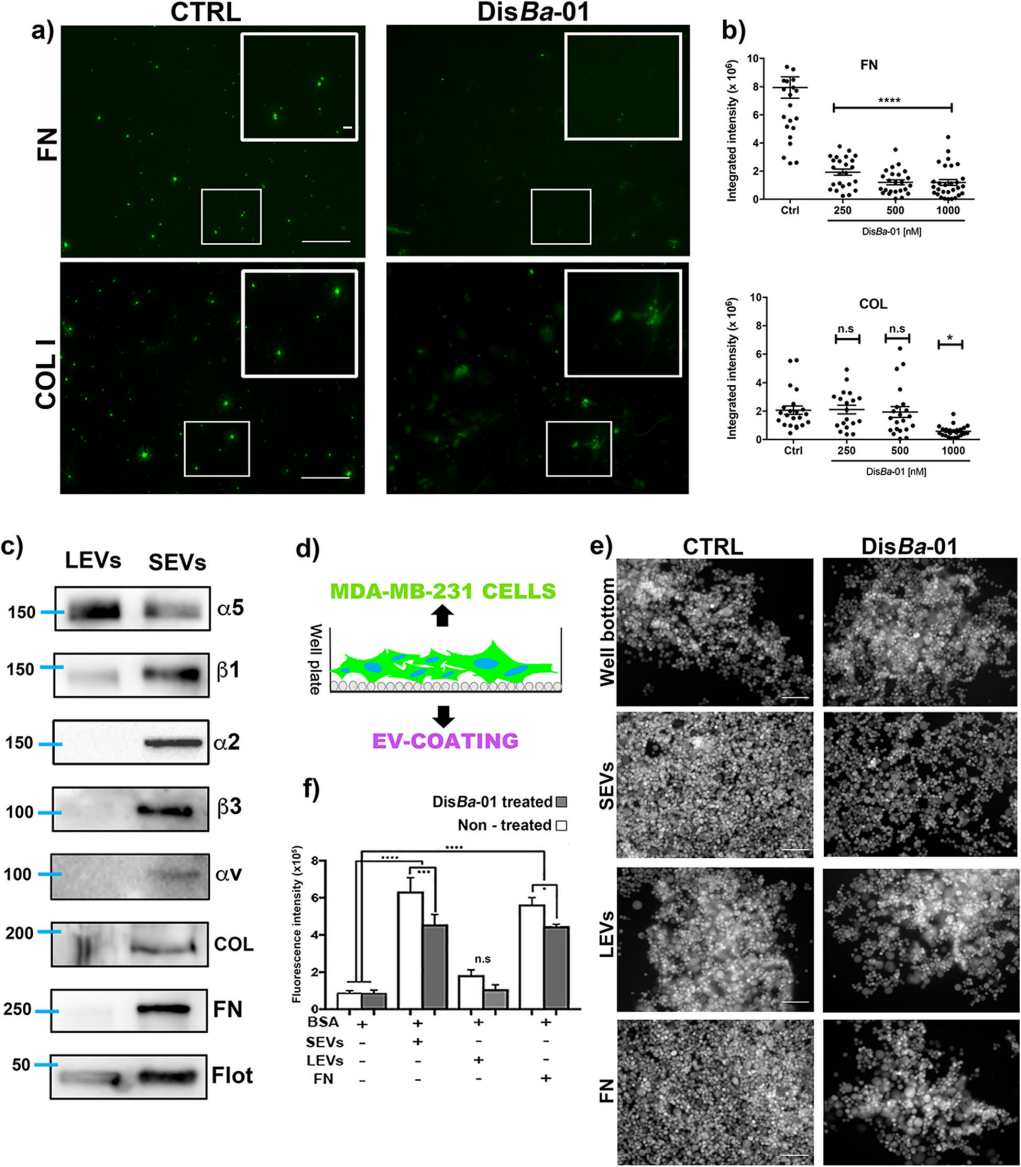
SEVs adhere to ECM components and support MDA-MB-231 cell adhesion. **a** Representative epifluorescence images of CFSE-labeled-SEV adhesion to fibronectin and collagen coating. Treated (Dis*Ba*-01500 nM) and untreated (CTRL) groups are compared. Scale bar: 100 μm (whole images); 10 μm (zoomed images). **b** Quantitation of SEV adhesion to fibronectin and collagen. **** *p* < 0.0001; * *p* < 0.05. **c** Western blot analysis of integrin subunits, α5, β1, α2, β3, and αv and ECM proteins, FN and COL on isolated SEVs and LEVs. **d** Schematic cartoon of cell adhesion assay to EV coating. **e** Representative images of Calcein AM-labeled MDA cell adhesion to well bottom, SEVs, LEV and FN coating comparing cell adhesion in control (CTRL) and Dis*Ba*-01 (100 nM) treated conditions. **f** Calcein AM fluorescence intensity of adherent cells. Comparison of adhesion in different coating and effect of Dis*Ba*-01 at 100 nM. * *p* < 0.05; *** *p* < 0.001; **** *p* < 0.0001

We found that SEVs adhere to both collagen I (COL I)- and FN-coated plates (Fig. 2a). The presence of Dis*Ba*-01 in concentrations relevant to its affinity for αvβ3 integrin (250–1000 nM) inhibited SEV binding to FN, suggesting the presence of αvβ3 integrin on the surface of SEVs while only the highest Dis*Ba*-01 concentration inhibited binding to COL I (Fig. 2a, b). Western blot analysis showed that α5, α2, αv, β1 and β3 integrin subunits are present in SEVs whereas LEVs contain only α5 and β1 integrin subunits (Fig. 2c). Notably, SEVs but not LEVs also had a significant amount of associated COL I and FN (Fig. 2c).

The presence of ECM components associated with SEVs suggested a mechanism by which SEVs may associate with cells via ECM-integrin complexes that might interact with cellular integrin receptors [36]. To test this possibility, we performed cell adhesion experiments to EV-coated surfaces. Isolated SEVs (Suppl. Fig. 1) or LEVs were used as substrates to support MDA-MB-231 cell adhesion (Fig. 2d). Calcein-labeled MDA-MB-231 cells showed higher adhesion to SEV-coated wells compared to non-coated wells. Cell adhesion to SEV coating was comparable with the positive FN-coated control, which could potentially be explained by the presence of FN or COL bound to integrin subunits on the SEVs. On the other hand, LEV coating did not support significant cell adhesion when compared with control (Fig. 2d and e). In parallel, we treated cells and SEV/LEV coating with Dis*Ba*-01 (30 min incubation) to analyze the effect of integrin inhibition. Cell adhesion on SEV and fibronectin coating was significantly reduced by Dis*Ba*-01 treatment, which was not observed for LEV coating (Fig. 2f).

### SEV uptake is inhibited by αvβ3 integrin blocking

**Fig. 3:**
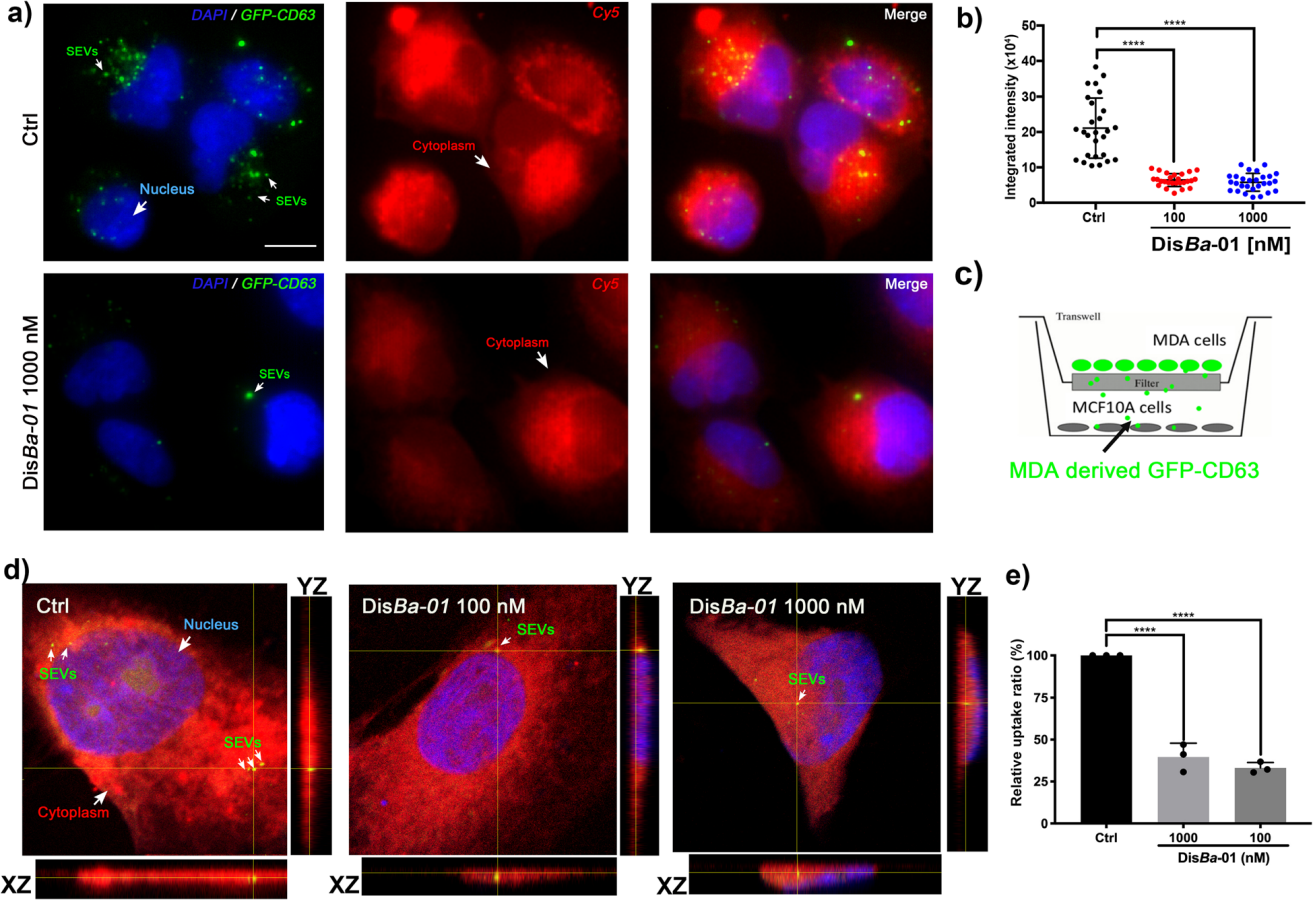
Inhibition of SEV uptake by αvβ3 integrin blocking. **a** Representative images of SEV uptake in MCF 10A cells labeled with cell tracker red. Arrows indicate internalized SEVs. Ctrl, control. Scale bar, 50 μm. **b** Integrated intensity of CFSE-labeled SEV internalized in MCF 10A cells; ****p < 0.0001. **c** Schematic view of transwell system. **d** Orthogonal view of MCF 10A co-cultured with MDA-MB-GFP-CD63 cells showing cells-derived GFP-CD63 uptake in control (Ctrl) and Dis*Ba*-01 (100 and 1000 nM) treated cells. Arrows indicate internalized GFP-CD63. Scale bar, 10 μm. **e** Quantitation of relative uptake of vesicles; ****p < 0.0001

An important question in the EV field is how are EVs recognized and taken up by recipient cells. On the basis of the adhesion data shown in Fig. 2, we performed immunofluorescence microscopy to investigate whether the interaction of EVs with recipient cells would be affected by Dis*Ba*-01. SEVs purified from MDA-MB-231 cells were labeled with carboxyfluorescein succinimidyl ester (CFSE), incubated with Dis*Ba*-01 in different concentrations for 30 min, and added to adherent non-tumorigenic MCF 10A breast epithelial cells. Epifluorescence microscopy revealed the presence of green fluorescent signals in MCF 10A cells suggesting the internalization of SEVs. On the other hand, treatment of SEVs with Dis*Ba*-01 (100 and 1000 nM) caused a significant reduction of this uptake (Fig. 3a and b).

To identify whether αvβ3 integrin plays a role in SEV uptake, we stably expressed GFP-CD63 in MDA-MB-231 cells. A tetraspanin CD63 is an intrinsic membrane protein that is involved in exosome biogenesis and detected only in the SEV fraction (Fig. 1c) being considered an exosome/SEV marker [46]. MDA-MB-231 cells expressing GFP-CD63 (donor cells) and MCF 10A cells (recipient cells) were respectively plated in the upper and lower wells of Transwell plates (Fig. 3c). GFP-CD63-enriched SEVs secreted from MDA-MB-231 cells were internalized into MCF 10A cells and the internalization was significantly reduced by Dis*Ba*-01 (Fig. 3d and e), suggesting that αvβ3 integrin has a key role in this uptake.

### Integrin inhibition affects uptake of tumor SEVs by breast epithelial cells

Thus far, our experiments show that adhesion and uptake of purified SEVs by MCF10A cells are reduced by Dis*Ba*-01 using a transwell co-culture system. To explore the role of αvβ3 integrin in the interchange of EVs between breast cancer and epithelial cells, MDA-MB-231 and MCF 10A cells were labeled with the cytoplasmic Cell Trace CFSE (Thermo Scientific) and Cell Tracker Red CMTPX (Thermo Scientific), respectively. After 24 h of co-culture, the transfer of vesicles from tumor cells to non-tumorigenic epithelial cells was clearly observed in the control condition by the presence of green signals in the red cells (Suppl. Fig. 2a). By contrast, we did not observe significant uptake of red signals by the green cells. Additionally, delivery of vesicles to MCF 10A cells was strongly reduced upon treatment for 24 h with 1000 nM of Dis*Ba*-01 (Suppl. Fig. 2b). To validate this finding, we used the GFP-CD63-expressing MDA-MB-231 cells in the adjacent co-culture model and a confocal microscopy to validate that the SEVs were inside of the recipient cells.

**Fig. 4:**
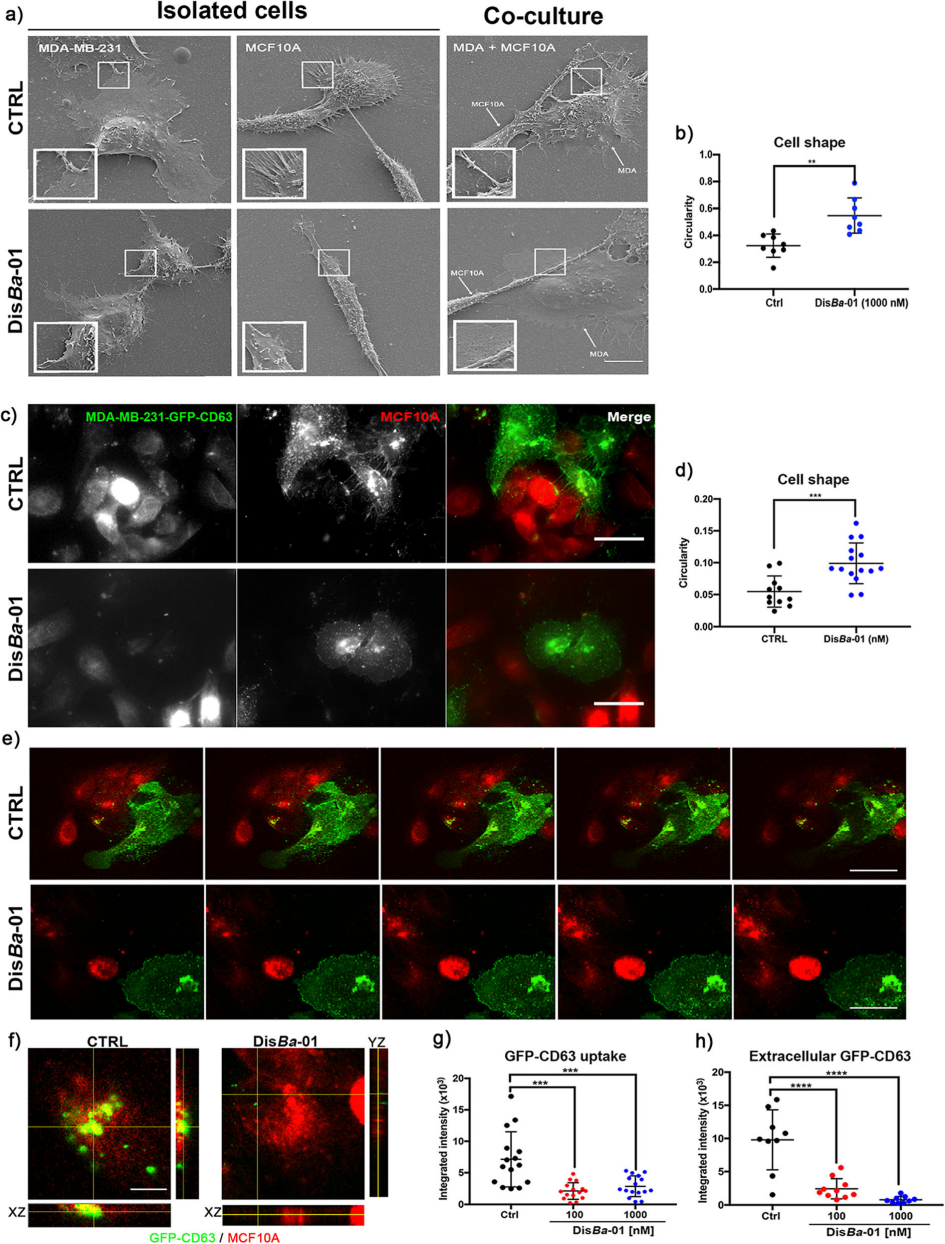
Dis*Ba*-01 inhibits SEV uptake in a co-culture system. **a** Scanning electron microscopy of MDA-MB-231 and MCF 10A cells in single and co-culture systems. Ctrl, control. Dis*Ba*-01, 1000 nM. Scale bar, 10 μm. **b** Effect of integrin inhibition on MDA-MB-231 cell morphology; **p < 0.01. **c** Representative epifluorescence images highlighting the distribution of GFP-CD63-enriched vesicles between MDA-MB-231 expressing GFP-CD63 (green) and MCF 10A (red) cells. Scale bar, 50 μm. **d** Effect of integrin inhibition on MDA-MB-231-GFP-CD63 cell morphology; ***p < 0.001. **e** Extracellular GFP-CD63-EVs from tumor to non-malignant cells. Z-stack slices from confocal acquisition show reduction of internalized and extracellular SEVs in Dis*Ba*-01 treated cells. **f** Orthogonal view of MCF 10A cells showing internalized GFP-CD63-SEVs and their reduction upon Dis*Ba*-01 treatment. **g** Effect of integrin inhibition on SEV uptake; ***p < 0.001. **h** Effect of integrin inhibition on extracellular GFP-CD63; ****p < 0.001

Analysis of scanning electron microscopy (SEM) confirmed the communication between tumor and non-tumorigenic cells (Fig. 4a, co-culture). Dis*Ba*-01 changed MDA-MB-231 cell morphology that was confirmed by circularity values of control and Dis*Ba*-01-treated cells (Fig. 4a, left column and b). In co-culture, the two different cell lines were connected to each other through either filopodia or retraction fibers and that connection was reduced by Dis*Ba*-01 (Fig. 4a, right column, zoom-in). Using fluorescence microscopy, we confirmed that GFP-CD63-enriched vesicles are transferred through the connection observed in Fig. 4a (Fig. 4c). MDA-MB-231-GFP-CD63 cell morphology was also affected by Dis*Ba*-01 (Fig. 4d). Confocal microscopy revealed a significant reduction of GFP signal in MCF 10A cells after Dis*Ba*-01 treatment (Fig. 4e, f, and g; Supplementary Movie S1). Furthermore, extracellular deposition of GFP-CD63 was also reduced by Dis*Ba*-01 (Fig. 4h).

### αvβ3 inhibition affects GFP-CD63 content in MDA-MB-231-GFP-CD63 cells

**Fig. 5:**
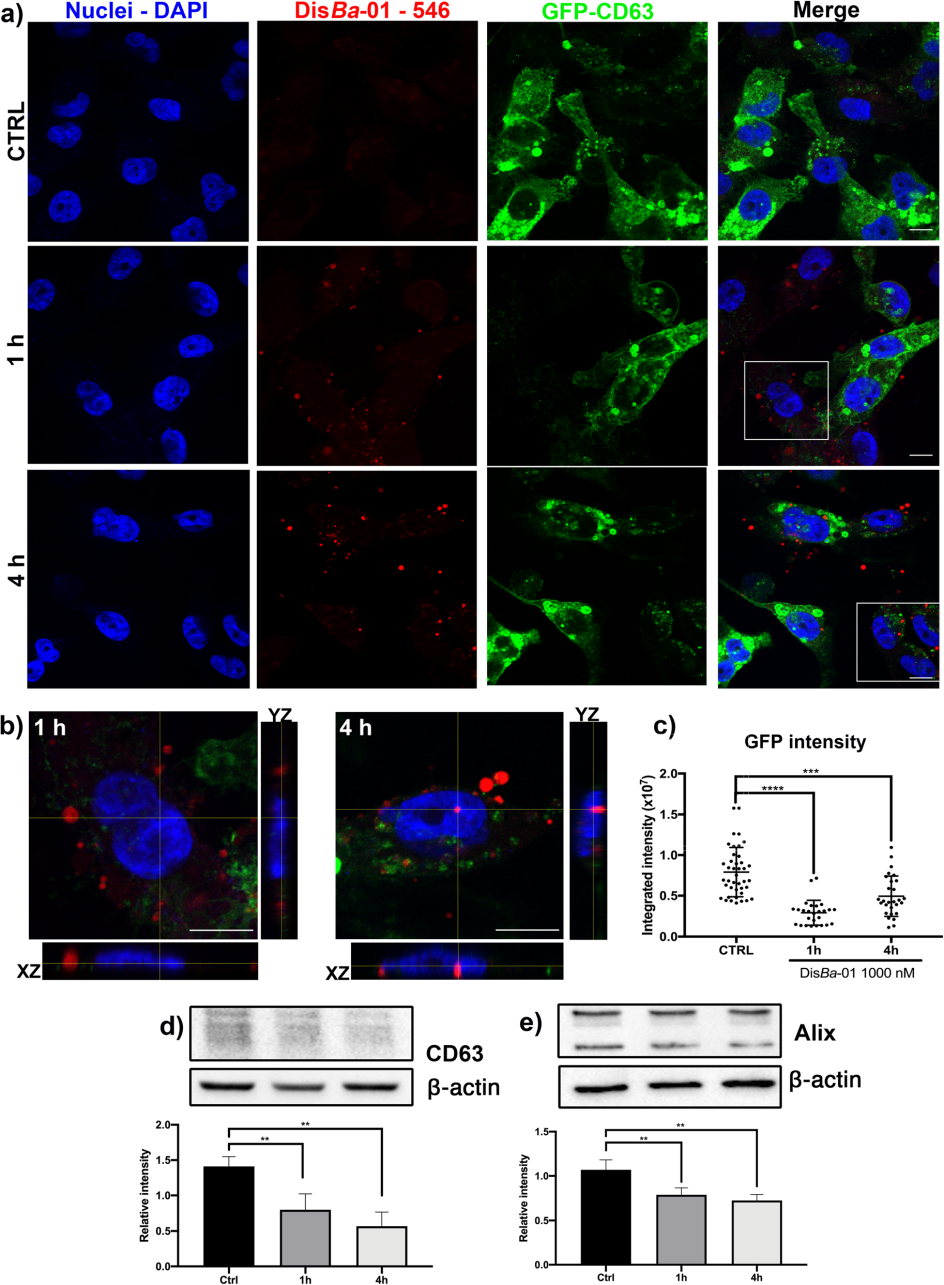
Dis*Ba*-01 decreases GFP-CD63 content. (**a**) Stacks from confocal images showing reduction of GFP-CD63 intensity 1 h and 4 h after Dis*Ba*-01 treatment. CTRL, control. (**b**) Orthogonal view of Dis*Ba*-01-Alexa Fluor-546 internalized on MDA-MB-231-GFP-CD63 cells, evidencing an increased level of red signal in 4 h condition. (**c**) Quantitation of green fluorescent signal of GFP-CD63. Reduced expression level of CD63 (**d**) and Alix (**e**) after treatment with Dis*Ba*-01 in western blotting analysis. **p < 0.01; ***p < 0.001; ****p < 0.0001. Scale bar, 10 μm

Considering the reduction of SEV uptake and extracellular CD63-GFP release by Dis*Ba*-01 treatment shown in Fig. 4, we investigated whether the disintegrin could affect SEV content inside the tumor cells. For this purpose, Alexa Fluor 546-labeled Dis*Ba*-01 was incubated with MDA-MB-231 cells expressing GFP-CD63 for 1 h and 4 h. Evident reduction of CD63 signal was observed in the treated cells at the two incubation times (Fig. 5a, b and c). The disintegrin was detected inside the cells after 1 h treatment and its signal was even stronger after 4 h, indicating internalization of the protein (Fig. 5b). Furthermore, we observed that the greater the disintegrin signal, the smaller was the GFP signal, which suggests that Dis*Ba*-01 could be altering the endogenous SEV biogenesis or content. To confirm this effect, we demonstrated by Western blotting of cell lysates that CD63 protein levels were reduced by treatment with Dis*Ba*-01 (Fig. 5d), corroborating the results obtained from confocal image analysis (Fig. 5c). Similarly, cellular levels of the exosome biogenesis-related protein Alix were also affected by disintegrin treatment (Fig. 5e). Since extracellular CD63 deposition was reduced by this treatment (Fig. 4h), these data suggest that binding of Dis*Ba*-01 with αvβ3 integrin may affect exosome biogenesis components in the endocytic system. Moreover, treatment of isolated vesicles with Dis*Ba*-01 reduced EV-αvβ3 integrin levels, supporting the effect of Dis*Ba*-01 on SEVs (Suppl. Fig. 4, c).

## Discussion

During tumor progression, constant exchange of information between cancer cells and the surrounding microenvironment must occur to support tumor growth, vascularization and spreading. Extracellular vesicles cooperate with these processes by delivering information from malignant to non-malignant cells and to the ECM [47]. The αvβ3 integrin participates actively in tumor development and has been extensively studied as a target for anticancer therapy at a cellular level [48–51]. However, the role of αvβ3 integrin in EVs has not been fully addressed. Here, we demonstrate the impact of αvβ3 integrin inhibition by the disintegrin Dis*Ba*-01 on tumor derived-SEV adhesion and uptake.

Exosomes are SEVs formed as intraluminal vesicles (ILVs) inside the lumen of endosomes during their maturation into late endosomes/multivesicular bodies (MVBs), in a process involving precise machineries, such as the endosomal sorting complex required for transport (ESCRT) [7, 52]. During this process, integrins trafficked to early endosomes can be sorted to late endosomes, packaged into ILVs and secreted as exosomal integrins [53, 54]. SEVs isolated from triple negative breast cancer cells contain αvβ3 integrin, which is the main target of Dis*Ba*-01. Dis*Ba*-01 inhibits cancer cell adhesion, migration and invasion as a result from its binding to αvβ3 integrin. The active binding site of αvβ3 integrin is recognized by the RGD motif within the amino acid sequence of Dis*Ba*-01, which impairs the interaction between the integrin and the ECM components, interfering in cell-microenvironment processes [55–58]. Therefore, we decided to use Dis*Ba*-01 to study the role of SEV-αvβ3 integrin.

Purified SEVs adhesion to FN coating on tissue culture plates was significantly inhibited by Dis*Ba*-01, which suggests that αvβ3 integrin is involved in the interaction between SEVs and ECM proteins [23]. We discarded a possible effect caused by the EV-marker in αvβ3 integrin, since CFSE chemistry is highly employed in cells and it is not toxic, being used in diverse EV-papers [59–62]. Moreover, the αvβ3 integrin is a transmembrane receptor, whose C-terminal end is located inside the membrane while its N-terminal is located outside the membrane. In this way, we would not expect a chemical interaction between the active CFSE and the αvβ3 integrin of EVs, attributing the observed effect to Dis*Ba*-01/EV- integrin binding.

We have also found that the adhesion of MDA-MB-231 cells to tissue culture plates was aided by SEVs but not by LEVs, which lack αvβ3 integrin and FN; both molecules were detected only in SEVs. Previous reports described the ability of tumor derived EVs in promoting tumor cell adhesion [33, 34]. Also, the role of EV-αvβ3 integrin in platelets adhesion has been demonstrated [63]. Upon Dis*Ba*-01 addition, cell attachment was inhibited only in SEV coating, corroborating that SEV binds to cells in an integrin-dependent manner and with the involvement of ECM components. However, the integrin-ECM interaction is a complex process, and additional elements could influence EV-αvβ3 integrin / cell / ECM communication in the tumor microenvironment. For example, integrins form complexes with other membrane receptors such as growth factor receptors and proteoglycans, and its inhibition triggers the activation and inhibition of different pathways, including integrin recycling [64, 65]. Likewise, the expression of other types of integrins can occur in order to recover cell adhesion upon its suppression by some inhibitors [64, 66, 67]. These alterations can result in different fates of EV on cells, thus, is crucial to understand all the machinery involved in EV interactions with ECM, cell and other microenvironment components to address the complete elucidation of the mechanism by which EV-integrins participate in tumor progression.

Surface ligands present on EVs are probably the main agents responsible for the specific targeting of EV [68]. For cancer cells, the transference of EV content can dictate the success of metastasis. In this context, the exosomal αvβ3 integrin is related to the propagation of integrin-associated migratory phenotypes to recipient non-tumor cells [30, 69–71], being a convenient EV-receptor for uptake experiments. We addressed the ability of Dis*Ba*-01 in reducing uptake of MDA-MB-231 cell-derived SEVs by MCF 10A cells. As expected, the amount of labeled SEVs in recipient cells was significantly reduced, indicating an active participation of αvβ3 integrin in this route. Furthermore, we designed a new transwell co-culture system using MDA-MB-231 cells stably expressing GFP-CD63 and detected the reduction of GFP-CD63-enriched exosome binding and internalization into MCF 10A cells. This result confirmed the participation of SEV- αvβ3 integrin in cell-cell communication.

Similar results were obtained when malignant cancer cells and non-malignant epithelial cells where co-cultured in the same compartment. Despite the absence of phenotypic alterations in non-malignant epithelial cells, αvβ3 integrin inhibition altered MDA-MB-231 cell morphology, reduced filopodia-like protrusions and vesicle delivery. Furthermore, quantitative analysis of confocal fluorescence images from MDA-MB-231-GFP-CD63 and MCF 10A co-culture showed that Dis*Ba*-01 treatment not only inhibited uptake of SEVs by recipient cells but also reduced the number of vesicles released to the extracellular space.

Dis*Ba*-01 internalization induced the downregulation of GFP-CD63 levels in donor cells, data supported by reduction of CD63 and Alix protein in cell lysates. CD63 and Alix are among the proteins mostly identified on exosomes. CD63 is a tetraspanin widely explored as exosome marker, as is expressed in various late endocytic organelles [72, 73], while Alix works as an auxiliary component for the ESCRT machinery during ILVs formation [74, 75]. Alix also binds to the cytosolic adaptor syntenin, which in turns connects to the transmembrane proteins syndecans and supports EV biogenesis [76]. Given the high affinity of Dis*Ba*-01 by αvβ3 integrin, the bound disintegrin could be internalized with the receptor, promoting effects on EV biogenesis. Besides, there is an association between integrins and syndecan proteins, and it is possible that an integrin inhibitor could affect syndecan functioning, impairing syndecan-syntenin-Alix complex formation and leading to imbalance of SEVs biogenesis. To the best of our knowledge, integrin regulation of EV biogenesis has not been reported yet. More in-depth investigations are necessary to understand how integrin inhibition affects the production and uptake of SEVs.

**Fig. 6:**
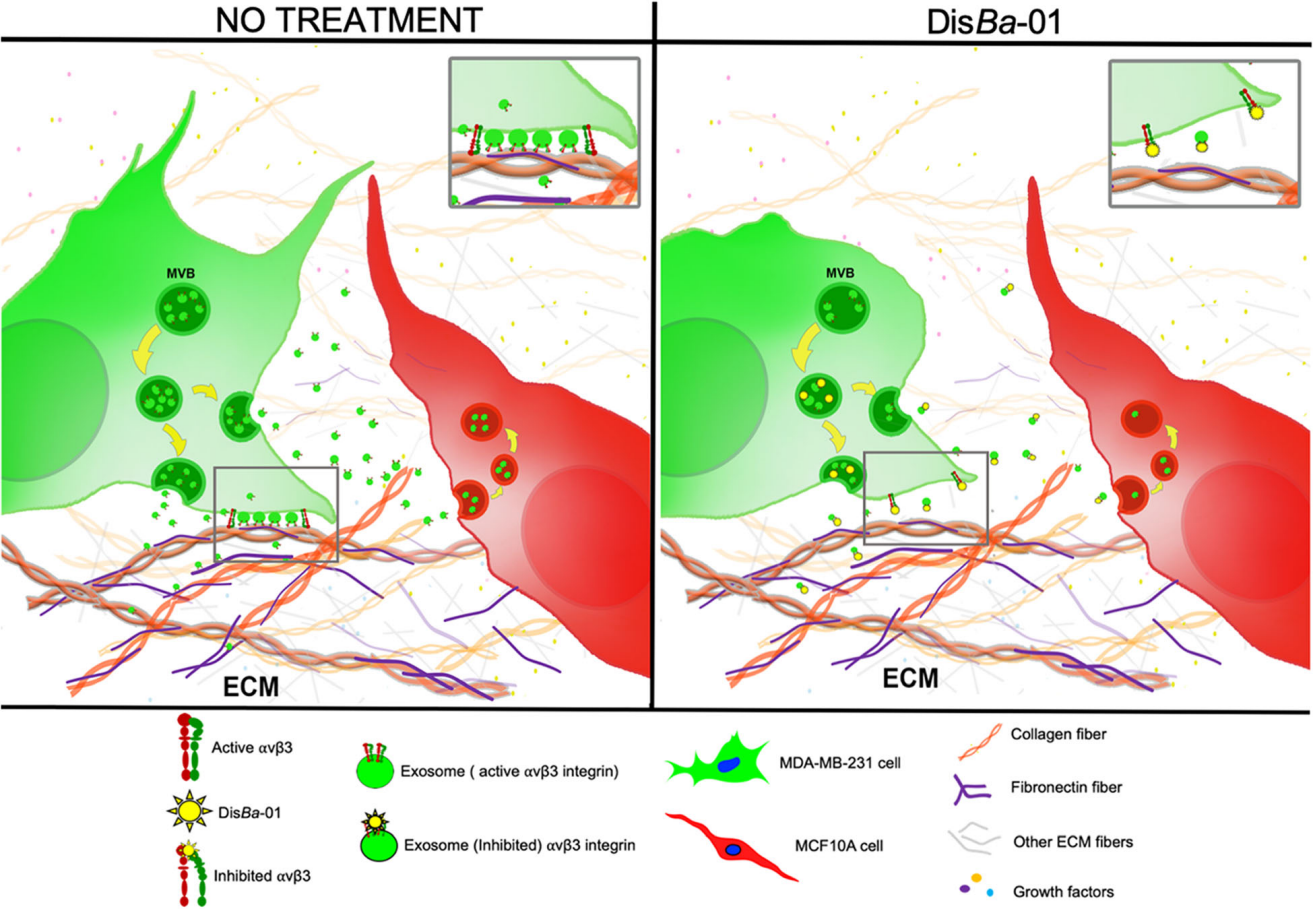
Proposed model for inhibition of SEV adhesion and cellular uptake by recipient cells upon blockage of αvβ3 integrin. Left panel: in the absence of Dis*Ba*-01, tumor cells secrete SEVs that bind to ECM and are taken by non-malignant cells. Right panel: blockage of αvβ3 integrin by Dis*Ba*-01 decreases SEV secretion, adhesion to ECM proteins and uptake by recipient cells

We propose a model of which Dis*Ba*-01 inhibits cell-cell communication by decreasing vesicle adhesion and transfer through binding to αvβ3 integrin in SEVs (Fig. 6), showing for the first time disintegrins acting in a vesicular level. Moreover, the results shown here highlight the relevance of αvβ3 integrin on a role of SEVs, which mediates cell-cell communication. Cancer cells can modify their environment by communicating with other cells through many mechanisms and cancer-derived EVs have been identified as a major way of cell communication [5, 6]. Mechanisms by which integrins in SEVs induce the interaction with recipient cells and how disintegrins inhibit this interaction are still unclear. Our results show that integrin inhibition is more complex than expected and may be helpful in defining new targets for cancer treatment, since there are no available pharmacological agents to modulate vesicular integrins from aggressive cancer cells [16].

## Conclusions

EVs are important players during tumor development, supporting cell communication with the microenvironment and adjacent cells. Here we provide evidence that adhesion receptors, formerly studied only at a cellular level, are present on the membrane of SEVs, thus being involved in processes such as EV adhesion and uptake. Our findings show that inhibition of αvβ3 integrin affects such processes, emphasizing the relevance of EV-carried integrins as a new target for cancer research. More in-depth research on the mechanisms should be addressed in future works.

## Supplementary information

**Additional file 1 : Supplementary Movie S1**. MCF 10A cell with internalized MDA-MB-231-GFP-CD63 content. Three-dimension projection from confocal imaging of MCF 10A (red) cell containing internalized GFP-CD63, related to Fig. 5. The movie was generated by Image J Fiji software from a Z-stack image (36 slices).

**Additional file 2 : Supplementary Figure S1**. Characterization of SEVs used for EV coating. (a) Representative trace and video acquisition snapshot from nanoparticle tracking analysis of small EVs obtained after an 18 h, 100,000 x *g* ultracentrifugation step. Traces show vesicles within a typical size profile. (b) Transmission electron microscopy of SEVs. Yellow arrows point to representative EVs. Scale bar: 500 nm (large image) and 100 nm (zoomed images). (c) WB for the EV markers CD63, Flotillin and Alix. WCL: whole cell lysate; UC-SEV: small extracellular vesicles from ultracentrifugation.

**Additional file 3 : Supplementary Figure S2.** MDA-MB-231 and MCF 10A cells EV exchange. Co-cultured cells labeled with cytoplasmic markers, showing exchange of EVs between cells. (a) Control conditions: MDA-MB-231 (green, left); MCF 10A (red, middle); MDA + MCF10A (right). (b) Treated conditions: MDA-MB-231 (Dis*Ba*-011000 nM, green, left); MCF 10A (Cell Tracker red, middle); MDA (Dis*Ba*-011000 nM) + MCF10A (right).

**Additional file 4 : Supplementary Figure S3.** Full-length blots related to the results presented in Figs. 1 and 2.

**Additional file 5 : Supplementary Figure S4.** Full-length blots related to the results presented in Fig. 5.

## Data Availability

The authors declare that the data generated in the current study are available within the article or from the corresponding author upon reasonable request.

## Abbreviations

CANX: Calnexin
CFSE: Carboxyfluorescein succinimidyl ester
DMEM: Dulbecco’s Modified Eagle Medium
ECM: Extracellular matrix
EVs: Extracellular vesicles
FBS: Fetal bovine serum
Flot: Flotillin
GFP: Green fluorescent protein
HS: Horse serum
K_d_: Dissociation constant
LEVs: Large extracellular vesicles
NTA: Nanoparticle tracking analysis
RGD: Arginine-Glycine–Aspartate
SEM: Scanning electron microscopy
SEVs: Small extracellular vesicles
TEM: Transmission electron microscopy
WB: Western blotting
WCL: Whole cell lysate
