# Supplementary figures and images for: Inhibition of αvβ3 integrin impairs adhesion and uptake of tumor-derived small extracellular vesicles
Source: Cell Commun Signal. 2020 Sep 25;18:158. doi: 10.1186/s12964-020-00630-w (PMC7520983; doi:10.1186/s12964-020-00630-w)

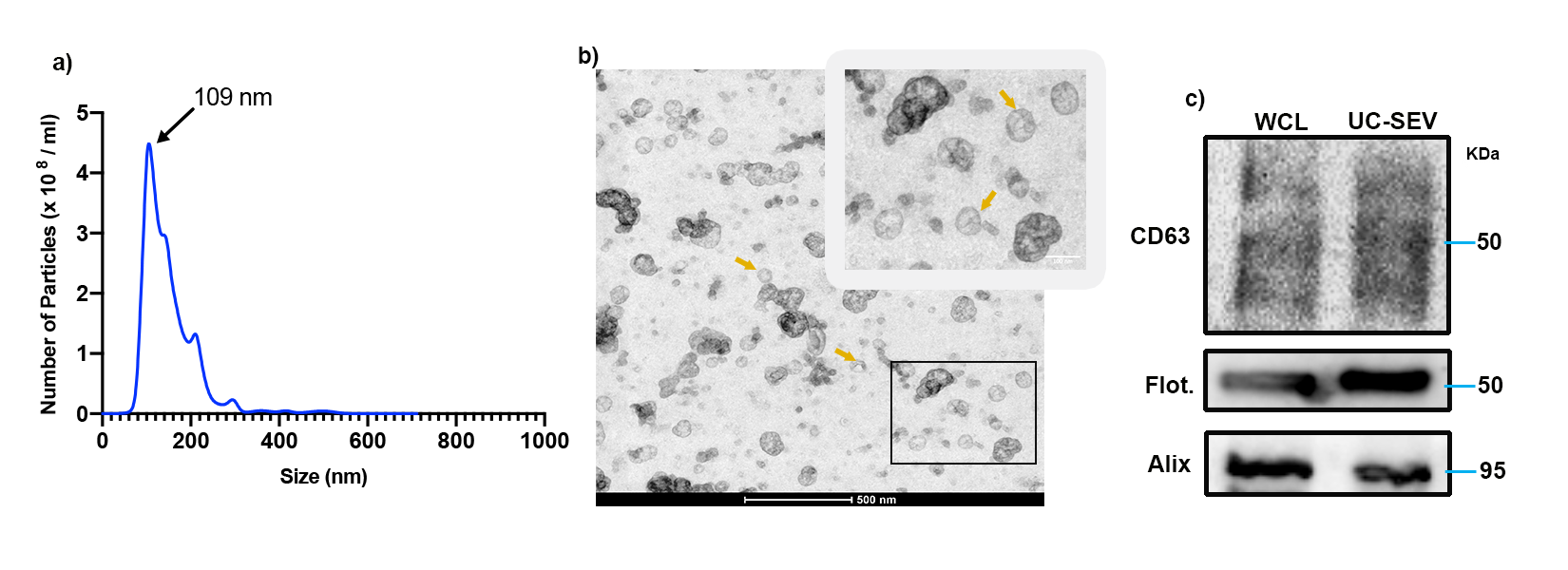

Supplement: Supplementary file 3 — Additional file 2 : Supplementary Figure S1. Characterization of SEVs used for EV coating. (a) Representative trace and video acquisition snapshot from nanoparticle tracking analysis of small EVs obtained after an 18 h, 100,000 x g ultracentrifugation step. Traces show vesicles within a typical size profile. (b) Transmission electron microscopy of SEVs. Yellow arrows point to representative EVs. Scale bar: 500 nm (large image) and 100 nm (zoomed images). (c) WB for the EV markers CD63, Flotillin and Alix. WCL: whole cell lysate; UC-SEV: small extracellular vesicles from ultracentrifugation. [file 12964_2020_630_MOESM3_ESM.png]

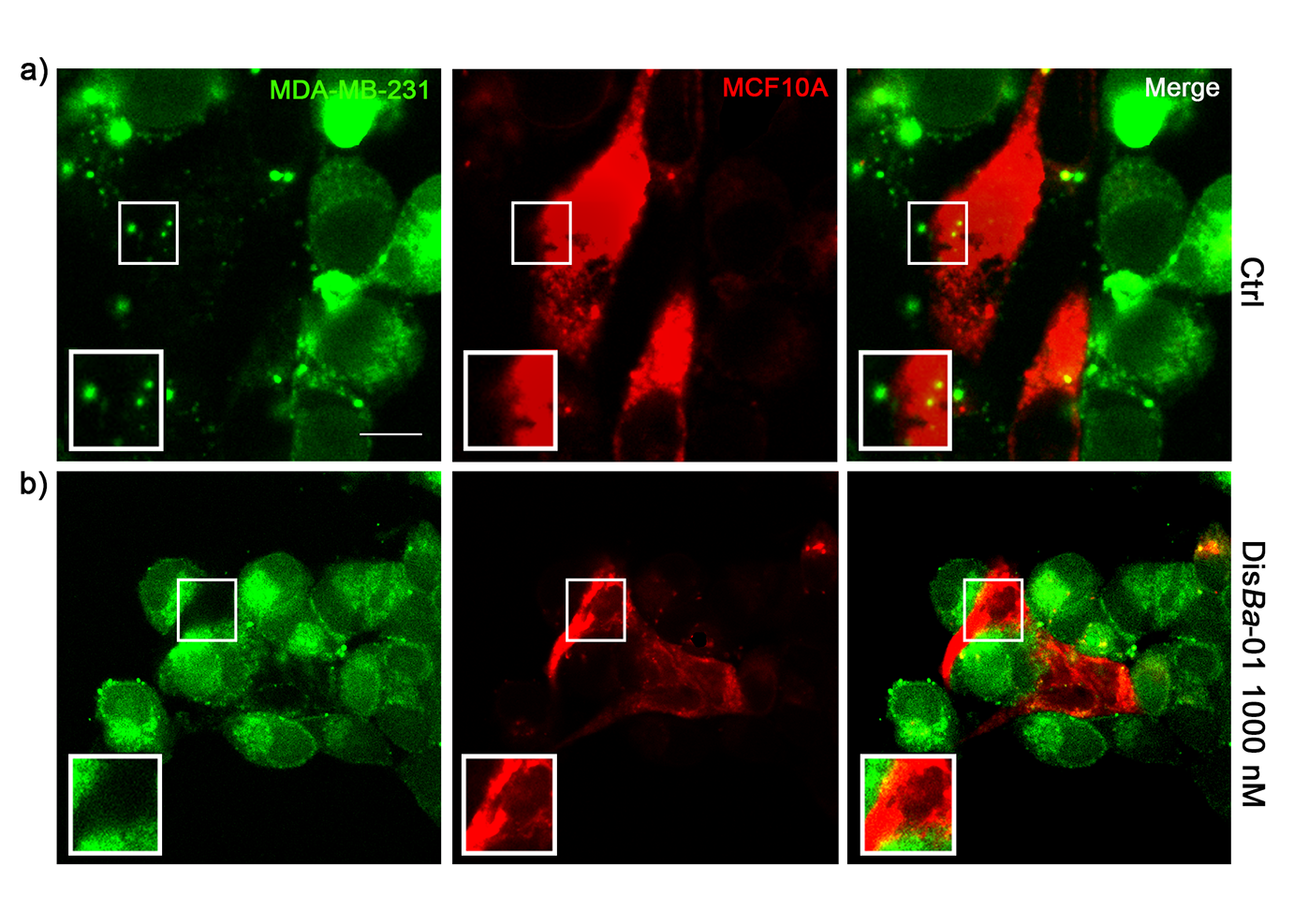

Supplement: Supplementary file 4 — Additional file 3 : Supplementary Figure S2. MDA-MB-231 and MCF 10A cells EV exchange. Co-cultured cells labeled with cytoplasmic markers, showing exchange of EVs between cells. (a) Control conditions: MDA-MB-231 (green, left); MCF 10A (red, middle); MDA + MCF10A (right). (b) Treated conditions: MDA-MB-231 (DisBa-011000 nM, green, left); MCF 10A (Cell Tracker red, middle); MDA (DisBa-011000 nM) + MCF10A (right). [file 12964_2020_630_MOESM4_ESM.png]

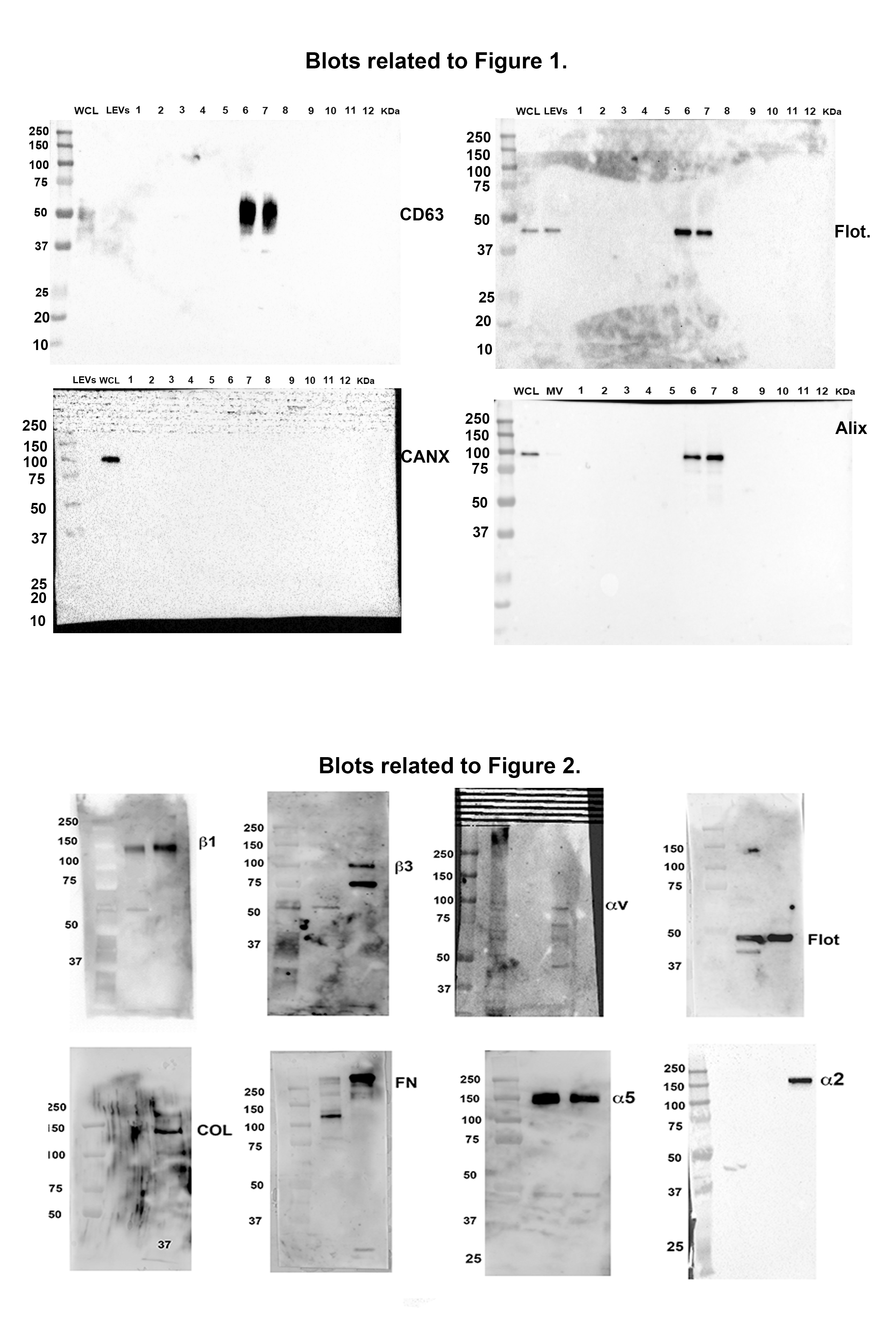

Supplement: Supplementary file 5 — Additional file 4 : Supplementary Figure S3. Full-length blots related to the results presented in Figs. 1 and 2. [file 12964_2020_630_MOESM5_ESM.png]

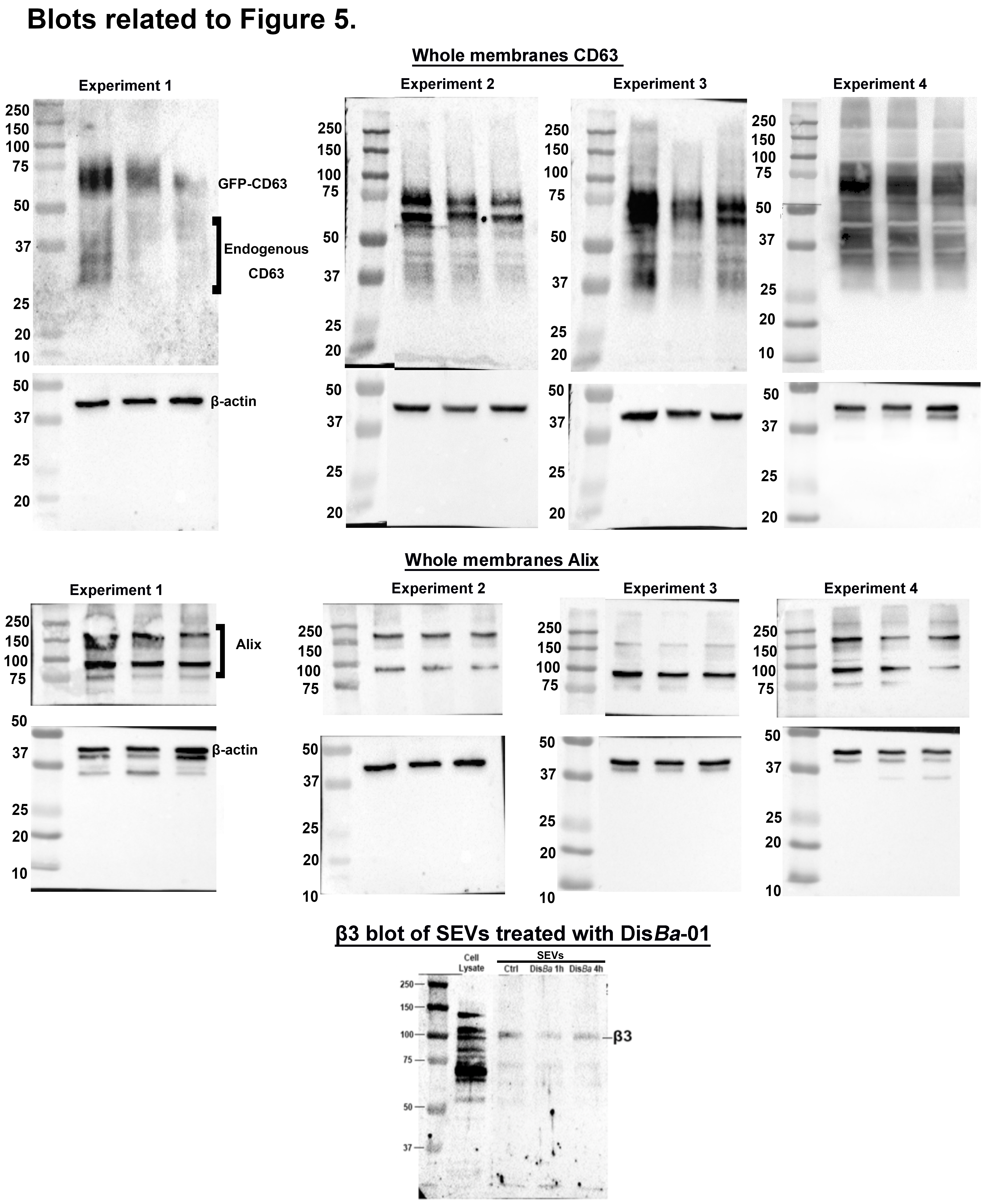

Supplement: Supplementary file 6 — Additional file 5 : Supplementary Figure S4. Full-length blots related to the results presented in Fig. 5. [file 12964_2020_630_MOESM6_ESM.png]
